# Increasing trends of lymphogranuloma venereum among HIV-negative and asymptomatic men who have sex with men, the Netherlands, 2011 to 2017

**DOI:** 10.2807/1560-7917.ES.2020.25.14.1900377

**Published:** 2020-04-09

**Authors:** Fleur van Aar, Michelle M Kroone, Henry JC de Vries, Hannelore M Götz, Birgit HB van Benthem

**Affiliations:** 1 Centre of Infectious Disease Control, National Institute for Public Health and Environment, Bilthoven, the Netherlands; 2 Department of Infectious Diseases, Public Health Service (GGD) Amsterdam, Amsterdam; 3 Department of Dermatology, Amsterdam Institute for Infections and Immunity (AI&II), Amsterdam University Medical Centres, Location Academic Medical Centre, Amsterdam, the Netherlands; 4 Department of Infectious Disease Control, Municipal Public Health Service, Rotterdam, the Netherlands

**Keywords:** Lymphogranuloma venereum, HIV infection, asymptomatic infection, epidemiology, Sentinel Surveillance, MSM

## Abstract

**Introduction:**

Lymphogranuloma venereum (LGV), an invasive form of *Chlamydia trachomatis* infection, has been reported among (mainly HIV-positive) men who have sex with men (MSM) since 2003. In the Netherlands, LGV testing recommendations changed from selective to universal testing in 2015. Changes in tested populations could have led to incomparable LGV positivity rates over time.

**Aim:**

We investigated LGV trends among MSM attending Centres for Sexual Health using surveillance data between 2011 and 2017.

**Methods:**

LGV positivity was calculated among MSM tested for rectal *Chlamydia* infection and MSM tested specifically for LGV. With multivariable logistic regression analysis, the association between years and LGV was adjusted for testing indicators and determinants.

**Results:**

We included 224,194 consultations. LGV increased from 86 in 2011 to 270 in 2017. Among LGV-positives, proportions of HIV-negative and asymptomatic MSM increased from 17.4% to 45.6% and from 31.4% to 49.3%, respectively, between 2011 and 2017. Among MSM tested for rectal chlamydia, LGV positivity increased from 0.12% to 0.33% among HIV-negatives and remained stable around 2.5% among HIV-positives. Among LGV-tested MSM, LGV positivity increased from 2.1% to 5.7% among HIV-negatives and from 15.1% to 22.1% among HIV-positives. Multivariable models showed increased odds ratios and significant positive associations between years and LGV.

**Conclusions:**

Although increased testing and changes in LGV incidence are difficult to disentangle, we found increasing LGV trends, especially when corrected for confounding. LGV was increasingly attributed to HIV-negative and asymptomatic MSM, among whom testing was previously limited. This stresses the importance of universal testing and continuous surveillance.

## Background

Lymphogranuloma venereum (LGV) is a sexually transmitted infection (STI) caused by invasive L1, L2 and L3 serovars of *Chlamydia trachomatis* (CT). Rectal LGV has been reported at a low level among men who have sex with men (MSM) in Europe since 2003, while urogenital infections are rarely seen [1-3]. Rectal LGV can cause proctitis or proctocolitis characterised by severe rectal pain, mucoid and/or haemorrhagic rectal discharge, tenesmus, diarrhoea or constipation and also systemic symptoms such as fever, malaise and weight loss. If left untreated, LGV proctitis can lead to chronic colorectal fistulas and strictures [4,5]. In addition, the ulcerative nature of LGV may increase the risk of STI and HIV transmission [6,7]. Differentiation of the LGV-causing L serovars from the non-LGV (D–K) serovars is important, as rectal LGV requires an extended doxycycline treatment regimen (21 days) compared with non-LGV rectal infection (7 days) [8,9].

To date, the exact LGV transmission dynamics are not well understood. A previous Dutch study found a rectal-urethral LGV ratio of 15:1 and therefore concluded that urethral LGV could not explain all rectal LGV transmissions [3]. Indications for tissue tropism have not been found. Urethral epithelium may be infected only briefly, long enough to contribute to ongoing transmission, but too short to become symptomatic and clinically detected [10]. This could explain why LGV is confined to networks of MSM engaged in high-risk sexual behaviours, including high rates of partner change. An oro-anal transmission route has been proposed to further explain the rectal-urethral LGV ratio [11].

Previous studies and the European Centre for Disease Prevention and Control underlined the importance of active LGV testing and the implementation of surveillance systems [4,7,12,13]. Still, surveillance programmes have not been implemented in many European countries owing to the limited availability of diagnostics [13]. In the Netherlands, LGV was added to the routine national STI surveillance system of Centres for Sexual Health (CSH) as part of the response to the outbreak in 2003. In addition, LGV testing recommendations were developed, which included rectal CT testing for only MSM who reported having had receptive anal sex. Among those with a positive rectal CT test result, LGV testing was recommended, in particular for HIV-positive MSM or MSM presenting with LGV-related symptoms.

Between 2006 and 2012, the number of LGV diagnoses fluctuated, but the incidence remained consistently higher than before the outbreak in 2003 most probably resulting from increased testing after 2003. However, it was estimated that selective LGV testing could have resulted in an underdiagnosis of 20% between 2006 and 2012, as LGV was also diagnosed among asymptomatic MSM and among HIV-negative MSM [12]. Therefore, the CSH testing recommendations changed in 2015 from selective to universal rectal chlamydia testing for all MSM and universal LGV testing in all rectal chlamydia-positive MSM [14].

Changes in the indications for LGV testing over time complicate the interpretation of LGV positivity trends (confounding by indication). A decreased positivity is to be expected when MSM at lower risk for LGV are increasingly tested, the incidence remains stable and LGV is truly uncommon in the population among whom testing was limited before. Because of selective testing procedures, positivity is usually calculated among only those positive for rectal CT who were further tested for LGV, thus excluding those negative for rectal CT [12]. In a universal testing situation however, the LGV positivity rate could also be calculated including rectal CT negatives into the denominator, which would provide a more representative estimate [15,16].

This study aimed to investigate rectal CT testing, LGV testing and LGV positivity using different denominators among MSM attending CSH between 2011 and 2017. In addition, we aimed to explore LGV trends with changes in the characteristics of the population tested for LGV taken into account.

## Methods

This surveillance study used routinely collected data from all consultations performed at CSH in the Netherlands between 2011 and 2017 [17].

### Data source and variables

The CSH offer low-threshold and free of charge STI/HIV care exclusively for predefined high-risk populations. MSM are eligible for STI/HIV testing independent of risk behaviour. A selection of information registered in the electronic patient records of the CSH, including demographic variables, sexual risk behaviour, STI/HIV history, STI testing and diagnosis, was sent to the CSH surveillance database for national surveillance purposes. For the current study, all MSM (men who had sex only with men and men who had sex with both men and women) consultations between 2011 and 2017 were included. Trends including earlier years have been published previously [12].

### Laboratory testing and case definition

Testing for LGV applied a two-step procedure. First, samples were tested by a generic nucleic acid amplification test (NAAT) for CT. Second, positive samples were further tested by a second PCR to discriminate between LGV and non-LGV genotypes. Only laboratory-confirmed LGV cases are reported to the surveillance system. Indeterminate LGV test results were considered as negative in the analyses.

### Data analysis

A client identification number was not available over the full study period and therefore the unit of analysis is ‘STI consultation’. Therefore, repeat testers could have been counted several times over the period analysed. We calculated the proportion of consulting MSM who were tested for rectal CT infection, the proportion of rectal CT-positive MSM among those tested and the proportion of MSM who were tested for LGV serovars among those with a rectal CT infection. LGV positivity was calculated by dividing the number of LGV-positives by the number of MSM who were (i) tested for rectal CT-infection, including those positive but not tested for LGV as ‘non-LGV’ and (ii) tested for LGV, thus excluding rectal CT-negative MSM. Proportions and positivity were stratified by year of consultation and by HIV status. All trends were explored using the using the Cochran–Armitage test for trends.

We performed uni- and multivariate logistic regression to adjust for possible confounders that could influence the association between year (categorical: 2011–17) and LGV. Indicators for LGV testing and variables that were previously identified as LGV risk factors using the same Dutch CSH surveillance data between 2006 and 2012, were added to the multivariate model: age group (< 35; 35–39; 40–44; ≥ 45 years), STI symptoms (no; yes; unknown), HIV status (negative; positive: known or new infections), sexual contact (sex with men only; sex with both men and women), number of sexual partners in the past 6 months (0–1; 2–5; 6–50; ≥ 51) and condom use in last contact (yes; no; unknown) [12]. Records with missing data were excluded from analysis.

### Ethical statement

Ethical committee approval was not sought since we used de-identified routinely collected surveillance data.

## Results

A total of 224,194 consultations among MSM were registered between 2011 and 2017. The median age was 36 years, 69.5% were ethnic Dutch, 14.1% were known HIV-positives, 20.5% had a history of STI, 83.8% had sex with men only, 52.9% had 0–5 sex partners in the past six months, 60.9% had unprotected sex with the most recent partner, 22.9% experienced STI related symptoms and 19.4% were notified for STI/HIV exposure (Table 1). The proportion missing values was low for all variables, with the highest proportion of 5.1% for condom use with last partner (Table 1). The number of consultations among MSM increased from 21,783 in 2011 to 45,553 in 2017 (Table 2). All characteristics changed significantly over time. The most notable changes between 2011 and 2017 included increasing proportions of: MSM 34 years and younger (from 41.7% (9,092/21,780) to 48.8% (22,221/45,552), while the proportions in older age groups decreased), MSM with an STI history (from 10.9% (2,383/21,783) to 25.5% (11,614/45,553)) and MSM who had between six and 50 partners (from 39.3% (8,192/20,863) to 47.4% (21,181/44,702), while proportions of people with 0–1 and 2–5 partners decreased; Supplementary Table S1). Unprotected sex with the most recent partner decreased from 73.3% (14,796/20,187) in 2011 to 62.6% (27,415/43,782) in 2017.

**Table 1:** Characteristics of MSM attending Centres for Sexual Health, the Netherlands, 2011–2017 (n = 224,194)

	n	%
Median age in years (Q1-Q3)	36 (27 - 47)	
Age group (years)		
≤ 34	102,769	45.8
35–39	27,095	12.1
40–44	25,112	11.2
≥ 45	69,210	30.9
Missing	8	0.0
Migration background^a^		
Ethnic Dutch	155,890	69.5
Eastern Europe^b^	5,071	2.3
Turkey^b^	2,988	1.3
Europe other	17,500	7.8
Asia^b^	13,066	5.8
Central and South America^b^	7,253	3.2
Suriname^b^	6,803	3.0
The Netherlands Antilles/Aruba^b^	4,723	2.1
Morocco and North Africa other^b^	2,896	1.3
Africa other^b^	2,250	1.0
North America and Oceania	2,943	1.3
Unknown	2,811	1.3
STI-endemic migration background		
No/unknown	179,144	79.9
Yes	45,050	20.1
HIV infection		
HIV-negative	190,557	85.0
Known HIV-positive	31,608	14.1
Newly diagnosed with HIV	2,029	0.9
Number of partners (in past 6 months)		
0–1	22,204	9.9
2–5	96,299	43.0
6–50	95,831	42.7
≥ 51	3,633	1.6
Missing	6,227	2.8
Sexual contact		
Men only	187,917	83.8
Both men and women	36,268	16.2
Missing	9	0.0
Sex work (in past 6 months)		
No	217,786	97.1
Yes	4,227	1.9
Missing	2,181	1.0
Client of sex workers (in past 6 months)		
No	215,588	96.2
Yes	5,809	2.6
Missing	2,797	1.2
Condom use most recent partner		
No	136,473	60.9
Yes	76,323	34.0
Missing	11,398	5.1
Previously diagnosed with an STI^c^		
No	166,850	74.4
Yes	45,992	20.5
Don't know	2,251	1.0
Missing	9,101	4.1
Notified for STI/HIV exposure		
No	179,804	80.2
Yes	43,454	19.4
Missing	936	0.4
STI symptoms^d^		
No	171,955	76.7
Yes	51,425	22.9
Missing	814	0.4

CSH: Centres for Sexual Health; HIV: human immunodeficiency virus; MSM: men who have sex with men; Q1: first quartile; Q3: third quartile; STI: sexually transmitted infection.

^a^ Migration background was based on the (self-reported) country of birth of either the CSH attendee or the CSH attendee’s parents.

^b^ Classified as areas with high STI/HIV endemicity.

^c^ Between 2011 and 2014: in the past 2 years. Since 2015: in the past year.

^d^ Self-reported; type of symptoms unknown.

**Table 2:** Rectal CT testing, rectal CT positivity and LGV testing among MSM attending Centres for Sexual Health, the Netherlands, 2011–2017 (n = 224,194)

Year	Consultations	Rectal CT test		Rectal CT-positive		LGV test	
	N (100%)	n	%	n	%	n	%
All MSM							
2011	21,783	16,902	77.6	1,547	9.2	1,187	76.7
2012	24,640	19,831	80.5	1,800	9.1	1,430	79.4
2013	27,497	22,774	82.8	1,857	8.2	1,560	84.0
2014	29,939	26,952	90.0	2,220	8.2	1,917	86.4
2015	34,442	32,653	94.8	2,499	7.7	2,219	88.8
2016	40,340	38,512	95.5	2,976	7.7	2,582	86.8
2017	45,553	43,873	96.3	3,130	7.1	2,826	90.3
HIV-negative MSM							
2011	17,717	13,212	74.6	1,008	7.6	717	71.1
2012	20,124	15,668	77.9	1,180	7.5	858	72.7
2013	23,072	18,740	81.2	1,289	6.9	1,031	80.0
2014	25,413	22,570	88.8	1,579	7.0	1,303	82.5
2015	29,657	27,930	94.2	1,871	6.7	1,628	87.0
2016	34,841	33,060	94.9	2,268	6.9	1,922	84.7
2017	39,733	38,106	95.9	2,431	6.4	2,162	88.9
HIV-positive MSM^a^							
2011	4,066	3,690	90.8	539	14.6	470	87.2
2012	4,516	4,163	92.2	620	14.9	572	92.3
2013	4,425	4,034	91.2	568	14.1	529	93.1
2014	4,526	4,382	96.8	641	14.6	614	95.8
2015	4,785	4,723	98.7	628	13.3	591	94.1
2016	5,499	5,452	99.1	708	13.0	660	93.2
2017	5,820	5,767	99.1	699	12.1	664	95.0

CT: *Chlamydia trachomatis*; HIV: human immunodeficiency virus; LGV: lymphogranuloma venereum; MSM: men who have sex with men.

^a^ Known and newly diagnosed HIV infections.

### Rectal CT and LGV testing

The proportion of MSM who were tested for rectal CT infection increased from 77.6% to 96.3% between 2011 and 2017 (Table 2), whereas the proportion of rectal CT-positive MSM decreased from 9.2% to 7.1%. Among rectal CT-positive MSM, the proportion tested for LGV increased from 76.7% to 90.3%. As all proportions were higher among HIV-positives, LGV testing increased more strongly among HIV-negatives. All trends were significant (p < 0.001).

### LGV

**Figure 1:**
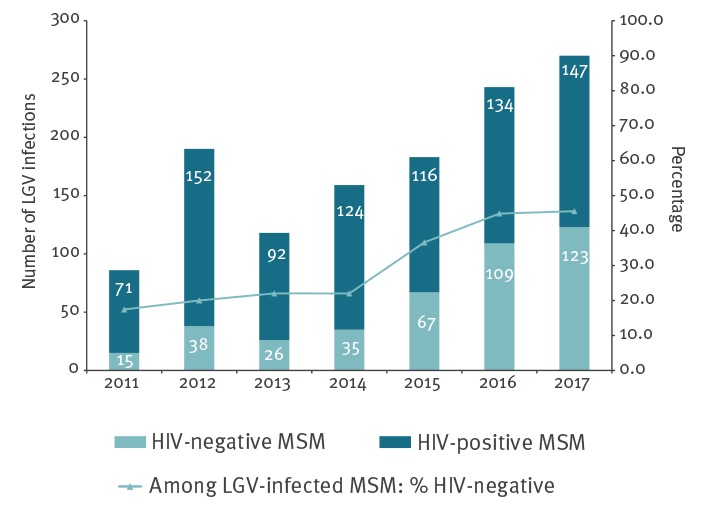
LGV among HIV-negative and HIV-positive MSM attending Centres for Sexual Health and proportion of HIV-negatives among all LGV cases, the Netherlands, 2011–2017 (n = 224,194)

HIV: human immunodeficiency virus; LGV: lymphogranuloma venereum; MSM: men who have sex with men.

The total number of diagnosed LGV infections increased from 86 in 2011 to 270 in 2017 (Figure 1). The proportion of LGV cases that were attributed to HIV-negative MSM increased from 17.4% (15/86) to 45.6% (123/270) between 2011 and 2017 (p < 0.001). The proportion of asymptomatic LGV infections increased from 31.4% (27/86) to 49.3% (133/270; p < 0.001). This proportion fluctuated among HIV-negative MSM (p = 0.658) but increased from 23.9% (17/71) in 2011 to 44.2% (65/147) in 2017 among HIV-positives (p < 0.001; Supplementary Table S2).

**Figure 2:**
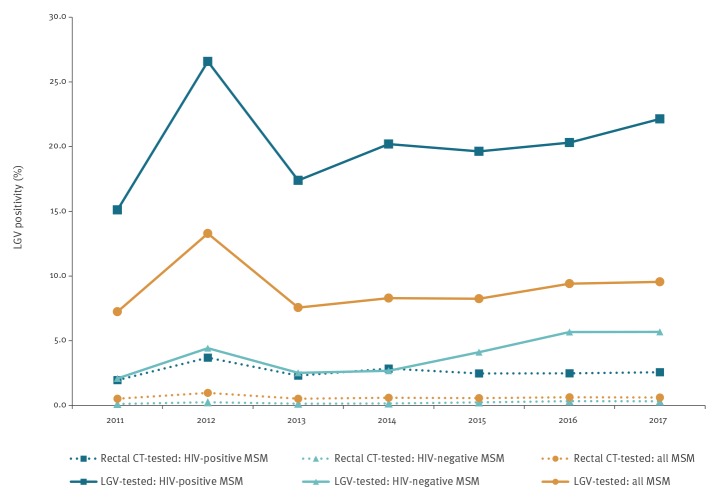
LGV positivity among all MSM and by HIV status for two different denominators: all MSM tested for rectal CT infection and MSM with positive rectal CT test and tested for LGV, the Netherlands, 2011–2017 (n = 224,194)

CT: *Chlamydia trachomatis*; HIV: human immunodeficiency virus; LGV: lymphogranuloma venereum; MSM: men who have sex with men.

Overall, LGV positivity was higher among HIV-positives than among HIV-negatives over the full study period, but the trends were comparable for both groups, with a rapid increase and decrease in 2012 and 2013, respectively (Figure 2). LGV positivity was low and stable around 0.6% (p = 0.232) when calculated among all MSM tested for rectal *Chlamydia* infection. Among HIV-positives the trend was stable around 2.5% (p = 0.534) and among HIV-negatives, the trend increased slightly but significantly from 0.12% (15/12,921) to 0.33% (123/37,837) p < 0.001). LGV positivity calculated among LGV-tested MSM was higher and increased from 15.1% (71/470) to 22.1% (147/664) among HIV-positive MSM (p = 0.340) and from 2.1% (15/717) to 5.7% (123/2,162; p < 0.001) among HIV-negative MSM.

**Figure 3:**
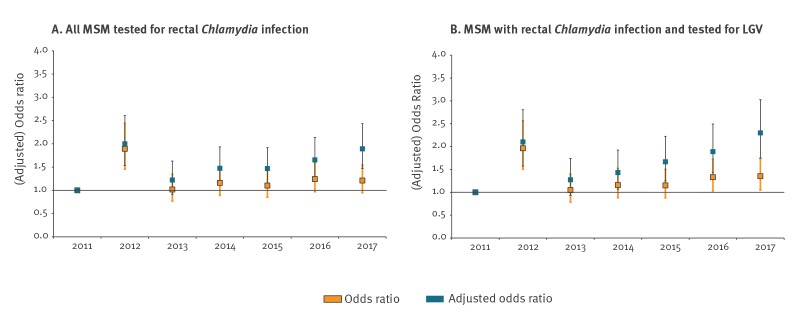
Odds ratios and adjusted odds ratios of the association between years and LGV, the Netherlands, 2011–2017 (n = 224,194)

LGV: lymphogranuloma venereum; MSM: men who have sex with men.

Figure 3 shows the results of the two logistic regression models for the association between years and LGV infection including rectal CT-tested MSM and LGV-tested MSM. In univariate logistic regression, LGV was significantly higher in 2012 compared with 2011 in both models. Including LGV-tested MSM only also resulted in significantly higher odds of LGV infection in 2016 and 2017. Correction for LGV testing indicators and risk factors resulted in higher adjusted odds ratios and significant positive associations between years and LGV infection in both models (except in 2013).

## Discussion

The Dutch CSH surveillance data showed that rectal CT testing, LGV testing and the number of LGV diagnoses increased among MSM between 2011 and 2017, with an increasing proportion of LGV attributed to HIV-negative or asymptomatic MSM. Given the increasing rectal CT and LGV testing trends, we expected positivity to decrease. In line with this, we found a decreasing trend for rectal CT. However, LGV positivity remained stable when calculated among all MSM tested for rectal CT infection and increased when calculated among LGV-tested MSM. These crude LGV positivity trends were affected by changes in the population tested over time, as we found stronger and significant positive associations between years and LGV after correction for LGV testing indicators and risk factors.

A strength of the current study is that CSH surveillance data have national coverage including all consultations at all CSH, resulting in a large number of observations. Unique is the collection of data on LGV since the first outbreak in 2003. Limitations of the current study include the lack of information on LGV-related symptoms, LGV risk factors such as unprotected receptive anal sex and LGV testing and diagnoses at healthcare settings other than CSH. In addition, we were not able to correct for repeat testing in the logistic regression analyses as a unique client identifier was not available for the full study period (available only July 2014–2017). However, the results of a generalised estimating equation model correcting for within-subject correlations, including the years 2015 to 2017 only, did not differ from the results of a logistic regression model (data not shown). Lastly, the interpretation of LGV positivity is challenging because changes in rectal CT positivity are also captured if LGV positivity is calculated among all MSM tested for rectal CT infection. Using LGV-tested MSM only as denominator, selection bias limits the interpretation of LGV positivity. Therefore, all trends and changes in the characteristics of the population tested should be considered.

Internationally, testing procedures vary from country to country and long-term surveillance data on LGV is lacking, which hampers comparison of trends between countries. The number of reported LGV cases continues to increase in European countries, which may be explained by increased testing [13]. In the United Kingdom, the reported numbers of LGV were higher than in the Netherlands and a similar overall upward trend with three rapid increases in 2005, 2010 and 2014 was observed [18]. In 2016, however, the UK reported a small decrease in the number of reported cases, which coincided with lower numbers of gonorrhoea and HIV infections among MSM. In the Netherlands, the increasing number of LGV infections coincided with increasing numbers and positivity rates of gonorrhoea and infectious syphilis [17]. A rising trend in the number of LGV infections was also observed in France, which was explained by a combination of improved surveillance, better access to testing and an increasing incidence possibly due to increases in condomless anal sex among MSM [19]. Outbreak investigations and surveillance data consistently showed that the majority of LGV cases occurred among HIV-positive MSM and that most of the cases presented with proctitis [2,4]. In contrast to previous reports showing that this profile of individuals with LGV has remained largely unchanged over the years, we found increasing proportions of HIV-negative and asymptomatic MSM among LGV-positives, up to almost 50% in 2017 [2,13,19,20].

The increasing proportions of HIV-negative MSM and asymptomatic cases among LGV-infected MSM could be explained by increased testing and previous underdiagnosis. Besides changes in the testing policy, the increasing proportion of HIV-negative MSM among LGV-positives may also be related to HIV biomedical interventions in two different ways: On the one hand, greater awareness of ‘undetectable is untransmittable’ for HIV and the availability of pre-exposure prophylaxis (PrEP) might have resulted in increased risky behaviour including sexual mixing between HIV-negative and HIV-positive MSM [21]. On the other hand, increasing STI trends could occur among HIV-negative MSM regardless of changes in behaviour, as it could also be explained by a decrease in HIV seroconversion among MSM using PrEP exhibiting high-risk behaviour [17,22]. With respect to asymptomatic LGV, our results showed an increase in the proportion of asymptomatic cases among HIV-positive MSM only, among whom LGV testing was already high in earlier years. Thus, the change in testing policy may not be the sole explanation. A hypothetical explanation could be a change in circulating LGV strains over time, as observed in France where the predominating strain changed from L2b to L2 between 2010 and 2015 [23]. Although not supported by French data, less severe clinical features or no symptoms have been observed among patients infected with the L2 strain compared with patients infected with the L2b strain in Spain [24].

In the Netherlands, LGV control is based on offering universal rectal CT testing and universal LGV testing to all MSM attending CSH, followed by treatment and (voluntary) partner notification for those with LGV. Although recommended by the Dutch guideline for general practitioners (GPs), rectal testing is rarely performed by GPs [25]. Therefore, insight into the occurrence of rectal CT and LGV among MSM attending other clinical settings in the Netherlands is needed, which would help to increase awareness of the current LGV epidemic among Dutch GPs. In general practice, HIV PrEP prescribing may provide an opportunity to increase rectal CT and LGV testing as part of the routine 3-monthly PrEP check-ups in a subgroup of high-risk HIV-negative MSM. In addition, asymptomatic infections deserve more attention, as an increasing number of asymptomatic infections may lead to delayed or missed diagnosis and consequently to increased LGV transmission. Another challenge of asymptomatic cases to LGV control is a longer recall period (6 months) for partner notification compared with symptomatic LGV (4–6 weeks before onset of symptoms) according to the partner management guideline for CSH [26]. In the literature, the relevance of detecting asymptomatic LGV is debated as a shorter course of doxycycline (7 days, similar to rectal *Chlamydia* infection) may effectively treat these infections. However, strong evidence is not available yet.

## Conclusion

It is challenging to disentangle the effects of increased testing, previous underdiagnosis and changes in LGV incidence over time. Current LGV control measures are mostly performed by CSH, which may not be enough to interrupt LGV transmission. To guide additional prevention efforts, insight into LGV occurrence among MSM attending clinical settings other than CSH and insight into circulating LGV strains are needed. Furthermore, it is important to take changes in the characteristics of the tested population into account in the interpretation of trends as we found stronger and significant increasing LGV trends when corrected for confounding. Notably, we found increasing proportions of LGV attributed to HIV-negative and asymptomatic MSM, among whom testing was previously limited. These findings stress the importance of universal rectal CT and LGV testing for MSM irrespective of behaviour, HIV status or symptoms and the importance of continuous surveillance to improve LGV control and to improve insight into the epidemic.

## Supplementary Data

107000 Supplement
