# Supplementary material for: Increasing trends of lymphogranuloma venereum among HIV-negative and asymptomatic men who have sex with men, the Netherlands, 2011 to 2017
Source: Euro Surveill. 2020 Apr 9;25(14):1900377. doi: 10.2807/1560-7917.ES.2020.25.14.1900377 (PMC7160438; doi:10.2807/1560-7917.ES.2020.25.14.1900377)
Supplement: Supplement [file 19-00377_Van-Aar_Supplement.pdf]

Supplementary material

This supplementary material is hosted by *Eurosurveillance* as supporting information alongside the article 'Increasing trends of lymphogranuloma venereum among HIV-negative and asymptomatic men who have sex with men in the Netherlands between 2011 and 2017' on behalf of the authors who remain responsible for the accuracy and appropriateness of the content. The same standards for ethics, copyright, attributions and permissions as for the article apply. *Eurosurveillance* is not responsible for the maintenance of any links or email addresses provided therein.

Table S1: Characteristics of MSM attending CSH by year of consultation between 2011 and 2017

	**2011 %**	**2012 %**	**2013 %**	**2014 %**	**2015 %**	**2016 %**	**2017 %**	*P* value[1]
Known HIV-positive	17.1	17.1	15.0	14.2	13.1	13.0	12.2	<0.001
STI/HIV endemic migration background[2]	17.1	18.6	18.4	19.3	20.5	21.6	22.2	<0.001
Age group								<0.001
≤34	41.7	42.4	44.1	44.8	47.2	47.7	48.8	<0.001
35-39	12.48	12.51	12.13	12.31	11.94	11.97	11.71	<0.001
40-44	14.04	13.48	12.42	11.15	10.56	10.01	9.45	<0.001
≥45	31.73	31.67	31.4	31.7	30.34	30.32	30.06	<0.001
Number of partners								
0-1	12.9	11.9	11.4	10.2	9.7	9.2	8.5	<0.001
2-5	46.2	45.3	45.5	45.3	44.2	42.9	42.2	<0.001
6-50	39.3	41.2	41.8	43.1	44.4	46.0	47.4	<0.001
≥51	1.6	1.6	1.4	1.4	1.7	1.9	1.9	<0.001
Unprotected sex most recent partner	73.3	73.4	65.9	58.6	59.9	62.1	62.6	<0.001
Asymptomatic	74.4	74.4	76.7	76.0	76.2	76.5	81.4	<0.001
STI/HIV notification	15.4	17.9	18.5	19.8	21.5	21.4	19.3	<0.001
Sex work	1.8	1.8	1.6	1.8	2.2	2.1	1.9	<0.001
Clients of sex workers	2.0	2.1	2.5	2.5	3.0	2.9	2.8	<0.001
Sex with men only	84.4	83.1	82.7	83.0	83.7	84.3	84.9	<0.001
Previously diagnosed with STI[3]	10.9	11.7	15.2	24.0	24.0	23.5	25.5	<0.001
Infectious syfilis[4]	2.0	2.1	2.1	2.3	2.6	2.9	2.6	<0.001
Gonorrhoea	9.0	9.3	9.3	9.5	10.8	11.3	11.0	<0.001
New HIV-infection	2.0	1.5	1.4	1.1	0.9	0.8	0.7	<0.001

Abbreviations: MSM: men who have sex with men; CSH: centres for sexual health; STI: sexually transmitted infection

1 Cochrane armitage trend test

2 STI endemic area's included: Eastern-Europe, Turkey, Asia, Central and South America, Caribbean and Africa. Migration background was based on (self-reported) the country of birth of both the CSH attendee and the CSH attendee's parents.

3 Between 2011 and 2014: in past 2 years. Since 2015: in past year.

4 Including primary, secondary and early latent syphilis

Table S2: Characteristics of LGV-positive MSM attending CSH by year of consultation

	**2011**	**2012**	**2013**	**2014**	**2015**	**2016**	**2017**	
N LGV-positive	86	190	118	159	183	243	270	
	%	%	%	%	%	%	%	*P* value[1]
HIV-negative	17.4	20.0	22.0	22.0	36.6	44.9	45.6	**<0.001**
Asymptomatic all MSM	31.4	33.2	26.3	39.6	47.0	40.9	49.4	**<0.001**
Asymptomatic HIV-negative MSM	66.7	42.1	34.6	65.7	62.7	45.4	55.7	0.658
Asymptomatic HIV-positive MSM	23.9	30.9	23.9	32.3	37.9	37.3	44.2	**<0.001**
STI/HIV endemic migration background[2]	25.6	18.4	21.2	23.3	24.0	25.1	28.2	**0.045**
Age group								
≤34	26.7	30.0	23.7	27.7	35.0	37.5	32.2	**0.035**
35-39	19.8	13.7	13.6	15.1	14.2	15.2	14.8	0.787
40-44	22.1	22.6	18.6	12.6	12.0	12.4	15.2	**0.003**
≥45	31.4	33.7	44.1	44.7	38.8	35.0	37.8	0.704
Number of partners								
0-1	0.0	2.8	4.5	3.3	2.3	4.7	2.6	0.396
2-5	34.2	32.4	28.6	26.6	31.1	27.2	26.7	0.123
6-50	63.4	61.0	60.7	63.6	62.7	61.6	65.0	0.541
≥51	2.4	3.9	6.3	6.5	4.0	6.5	5.6	0.249
Unprotected sex	91.4	85.3	84.2	75.3	76.0	68.6	71.5	**<0.001**
STI/HIV notification	30.2	28.4	30.5	40.3	39.9	37.9	28.2	0.556
Sex with men only	100.0	95.3	93.2	96.2	95.6	97.5	96.3	0.808
Previous STI diagnosis[3]	11.6	15.3	35.3	59.5	52.0	55.1	56.0	**<0.001**

Abbreviations: LGV: lymphogranuloma venereum; MSM: men who have sex with men; CSH: centres for sexual health; STI: sexually transmitted infection

1 Cochrane armitage trend test

2 STI endemic area's included: Eastern-Europe, Turkey, Asia, Central and South America, Caribbean and Africa. Migration background was based on (self-reported) the country of birth of both the CSH attendee and the CSH attendee’s parents.

3 Between 2011 and 2014: in past 2 years. Since 2015: in past year.
